# Antimicrobial Properties of an Immunomodulator - 15 kDa Human Granulysin

**DOI:** 10.1371/journal.pone.0156321

**Published:** 2016-06-08

**Authors:** Hung-Mu Wei, Li-Chih Lin, Chiu-Feng Wang, Yi-Jang Lee, Yuan-Tsong Chen, You-Di Liao

**Affiliations:** 1 Department of Biomedical Imaging and Radiological Sciences, National Yang-Ming University, Taipei 112, Taiwan; 2 Institute of Biomedical Sciences, Academia Sinica, Taipei 115, Taiwan; 3 Program in Molecular Medicine, National Yang-Ming University and Academia Sinica, Taipei, Taiwan; Nanyang Technological University, SINGAPORE

## Abstract

Granulysin, a cationic protein expressed by human natural killer cells and cytotoxic T lymphocytes, is a mediator for drug-induced Stevens-Johnson syndrome and graft-versus-host disease. Some 15 kDa granulysin are processed into 9 kDa forms and sequestered in cytolytic granules, while others are constitutively secreted into body fluids. Both 9 and 15 kDa granulysin have been shown to be a serum marker for cell-mediated immunity. Furthermore, 15 kDa is able to activate monocyte differentiation. However, its antimicrobial properties have not been clearly addressed. Here, we report a novel method to prepare both the soluble 9 and 15 kDa granulysin and show that the 15 kDa form is more effective than the 9 kDa form in exerting specific antimicrobial activity against *Pseudomonas aeruginosa* within a range of few micromolars. We also show that the 15 kDa granulysin is able to hyperpolarize the membrane potential and increase membrane permeability of treated bacteria. Interestingly, the bactericidal activity and membrane permeability of the granulysins were markedly reduced at lower pH (pH 5.4) as a result of probable increase in hydrophobicity of the granulysins. Additionally, we’ve also shown the granulysin to inhibit biofilm formation by *P*. *aeruginosa*. These results suggest that the 15 kDa granulysin exhibits a novel mechanism in bacteria killing in a way that’s different from most antimicrobial peptides. Our novel granulysin preparation methodology will be useful for further study of action mechanisms of other antimicrobial, cytotoxic and immunomodulating properties in granulysin-mediated diseases.

## Introduction

Worldwide emergence of multiple-drug-resistant (MDR) bacteria has led to the urgent need for the development of new antibiotics [1]. Antimicrobial peptides and proteins (AMPs) are important components of the host innate defensive system that inhibits invading pathogens [2–4]. As a result, AMPs are considered to be potent alternatives to conventional antibiotics. Although AMPs possess diverse secondary structures, their surfaces are amphipathic with cationic and hydrophobic residues on opposite sides within a hydrophobic environment. These AMPs have various modes of actions that differ from conventional antibiotics [4–6]. The positive-charged residues of AMPs promote selectivity for negatively charged components on microbial surfaces, whereas the hydrophobic regions of AMPs facilitate the interactions with the bacterial membrane. Disruption of membrane integrity and subsequent condensation of cytoplasmic components usually occur in the AMP-treated bacteria while inhibition of intracellular components can also occur without membrane damage [4, 7]. Various targets of AMPs have been extensively proposed, such as the outer surface lipid, outer membrane protein, inner membrane, inner membrane protein, intracellular protein, and nucleic acids [7–10].

Granulysin is a cytotoxic and proinflammatory protein produced by the human cytolytic T-lymphocytes and natural killer cells [11]. It is co-expressed with perforin and granzymes in cytolytic granules and released via receptor-mediated granule exocytosis [12]. The 9 kDa granulysin is derived from a 15 kDa precusor by truncation of both the N- and C-termini [13]. The recombinant 9 kDa granulysin has been shown to kill a variety of microbes such as bacteria, fungi, yeast, parasites and several tumor cell lines [11]. It can also kill extracellular *Mycobacterium tuberculosis* (MTB) by inducing lesions on the cell surface and damaging the intracellular MTB with perforin [12]. It also acts as a chemo-attractant for T lymphocytes, monocytes, and other inflammatory cells, and activation function in the expression of a number of cytokines, including RANTES/CCL5, MCP-1, MCP-3, MIP-1α/CCL3, IL-10, IL-1, IL-6 and IFN-α [14]. Additionally, it has been shown to be relevant to other clinical diseases including infection, cancer, transplantation, autoimmunity, skin afflictions and reproductive complications [11]. These reports reveal that granulysin plays an important role in immunomodulation and diseases, and may potentially be a therapeutic target. However, most of the studies have focused on the 9 kDa form instead of the 15 kDa form with the exception that the 15 kDa form has been shown to be an important mediator of drug-induced Stevens-Johnson syndrome and graft-versus-host disease (GVHD) [15–18].

Although the 9 kDa granulysin prepared by denaturation and refolding has been shown to exert antimicrobial activity on Gram-positive bacteria, such as *Staphylococcus aureus* and *Listeria monocytogenes*, and Gram-negative bacteria, such as *Escherichia coli* and *Salmonella typhimurium*, at the concentration of 10–25 μM [12, 19–21], large-scale production of bioactive soluble protein is still not successfully available due to bactericidal activity and formation of inclusion bodies in *E*. *coli* [13, 19, 22]. Furthermore, the 15 kDa form is generally regarded as a precursor form without much cytotoxicity against bacterial and mammalian cells [23, 24]. The commercially-available granulysin containing tags at the termini may be detrimental to protein function [25]. In this study, we have successfully developed an over-expression system in *E*. *coli* to produce soluble and bioactive recombinant 15 and 9 kDa forms of granulysin without denaturation/refolding procedures. The 15 kDa granulysin exerts antimicrobial activity preferentially against *Pseudomonas aeruginosa* and alters the membrane potential and permeability, but does not disrupt structural integrity. However, the antimicrobial activity is affected by salts, divalent cations and changes in pH. Additionally, it also inhibits the viability and formation of biofilms by *P*. *aeruginosa*. These results may benefit studies on bactericidal activity and cytotoxicity mediated by granulysin.

## Materials and Methods

### Cloning, expression and purification of human granulysin

The cDNAs encoding the 15 kDa human granulysin (Asn1-Leu130 from 520 mRNA transcript) and 9 kDa granulysin (Gly1-Arg74) [16] were tagged with DNA fragments containing *Hin*dIII-tagged PreScission^™^ Protease (GE Healthcare, Buckinghamshire, UK) recognition sequence (CTGGAAGTTCTGTTCCAGGGGCCC) and *Xho*I at the 5’ and 3’ends, respectively, and cloned into pGEM-T (Promega, WI, USA). In addition, DNA fragments containing His×6 peptide and maltose-binding protein (MBP) sequence were also constructed in pGEM-T with *Nde*I and *Hin*dIII at the 5’ and 3’ends, respectively. Both fragments were subsequently subcloned into the expression vector pET-22b (Merck Millipore, Darmstadt, Germany) through the *Nde*I/*Hin*dIII and *Hin*dIII/*Xho*I sites. Both 15 kDa and 9 kDa granulysins were expressed in *E*. *coli* BL21-CodonPlus(DE3)-RIL (Agilent, CA, USA) at 30°C overnight in the presence of 0.5 mM isopropyl-β-D-thiogalactopyranoside (IPTG). The crude cell lysate was passed through phosphate cellulose chromatography (P11, Whatman, Kent, England) using a 0.2–1 M NaCl gradient in 20 mM Tris-HCl, pH 7.4 and further purified by a HisTrap^™^ HP column chromatography (GE Healthcare, Uppsala, Sweden) using a 20–250 mM imidazole gradient in 20 mM HEPES, pH 7.4, 2 M NaCl. The granulysin in the soluble fraction was released and separated from maltose-binding protein by PreScission^™^ Protease and fast protein liquid gel filtration chromatography (FPLC Superose^™^ 12, GE Healthcare, Uppsala, Sweden) in 20 mM HEPES, pH 7.4, 0.15 M NaCl, 100 mM imidazole. The granulysins were further purified to homogeneity by HiTrap^™^ SP FF (GE Healthcare, Uppsala, Sweden) cation-exchange column chromatography using 0.15–1 M NaCl gradient in 20 mM HEPES, pH 7.4, and finally dialyzed against phosphate-buffered saline (PBS), pH 7.4, and stored at -70°C before use. The molecular masses of 9 and 15 kDa granulysins were determined by direct nanospray infusion of protein solutions. The isotopically resolved spectra acquired from orbitrap were further deconvoluted with the Xtract algorithm to determine the molecular weight [26].

### Antimicrobial Activity Assay

The Gram-negative bacteria *Escherichia coli* K-12 (M61655), *Pseudomonas aeruginosa* PAO1 (ATCC BAA-47^™^), *Klebsiella pneumoniae* (ATCC 13884), *Salmonella typhimurium* (ATCC 14028), *Yersinia enterocolitica* (ATCC 23715), *Serratia marcescens* (ATCC 8100) were separately cultured in Luria-Bertani broth (Merck Millipore, Darmstadt, Germany) and plated on Luria-Bertani agar. The Gram-positive bacteria *Listeria innocua* (ATCC 33090), *Staphylococcus aureus* (ATCC 6538P) and *Enterococcus faecalis* (ATCC 29212) were cultured and plated in tryptic soy broth/agar (BD, MD, USA). The bacteria were grown overnight, washed, and diluted 1:500 in 10 mM sodium phosphate, pH 7.5. Forty five μL of bacteria (ca. 1×10^5^ colony-forming unit (cfu)) were mixed with 5 μL of granulysin and incubated at 37°C for 3 hr. Serial dilutions of each granulysin-treated bacteria were prepared and plated for the determination of the remaining cfu. At least three independent experiments were performed for each assay to determine the average value with standard deviation.

### Confocal laser scanning microscopy (CLSM) and transmission electron microscopy (TEM)

*P*. *aeruginosa* PAO1 cells (ca. 1×10^7^ cfu) were cultured in an 8-well Lab-Tek^™^ II Chamber Slide^™^ (Thermo, MA, USA) and incubated with 2 μM 15 kDa granulysin at 37°C for 180 min in 10 mM sodium phosphate, pH 7.5. The cells were fixed with 4% paraformaldehyde, permeabilized by 0.5% Triton X-100, and incubated with anti-granulysin antibody RF10 (MBL, Chicago, USA) (1:1000) and Alexa Fluor 488-conjugated goat anti-mouse secondary antibody (Molecular Probes, OR, USA) (1:1000) for the localization of granulysin. Propidium iodide (Sigma, Missouri, USA) (1:1000) was also employed to stain bacterial DNAs inside the cytosol. After washing with PBS-T (PBS with 1% Tween 20), the images for granulysin (green) and DNA (blue) were taken by LSM 700 confocal laser-scanning microscope (Carl Zeiss, Jena, Germany) and merged [27]. The bacterial cells (ca. 1×10^7^ cfu in 95 μL) were incubated with 2 μM 15 kDa granulysin at 37°C for 3 hr in 10 mM sodium phosphate, pH 7.5, fixed by 4% paraformaldehyde and 1% osmium tetraoxide in 0.1 M cacodylate buffer, pH 7.4, and embedded in epoxy resin. Thin sections were double-stained with uranyl acetate and lead citrate, and the morphologies were observed under JEM 1200-EX transmission electron microscope (JEOL, Tokyo, Japan) [28].

### Membrane potential and permeability assays

*P*. *aeruginosa* PAO1 cells were collected from mid-log-phase culture, washed in HEPES buffer (5 mM HEPES, pH 7.2, and 20 mM glucose), and re-suspended in the same buffer with the addition of 0.2 mM EDTA to an OD_600_ of 0.05. The bacteria were incubated with 0.4 μM DiSC_3_(5) (3,3’-dipropylthiadicarbocyanine iodide, Molecular Probes, OR, USA) in the dark for 2 hr at room temperature with gentle agitation (150 rpm). The osmotic gradient was equilibrated to a final concentration of 0.1 M KCl. The granulysin and SMAP-29 were added to a 200 μL cell suspension in a High Precision Cell cuvette (Hellma Analytics, Mülheim, Germany). The fluorescence intensity was determined by an FP-8500 fluorescence spectrophotometer (Jasco, Tokyo, Japan) with an excitation wavelength of 622 nm and an emission wavelength of 670 nm [28]. Alternatively, the membrane potential of bacteria was also determined by the *Bac*Light^™^ Membrane Potential Kit (Molecular Probes, OR, USA) as described by the manufacturer. Microbes were collected, washed and re-suspended to an concentration of 1×10^6^ per mL in PBS and stained with 30 μM DiOC_2_(3) (3,3’-diethyloxa-carbocyanine iodide) for 5 min. The samples were then incubated with antimicrobial agents for 30 min at room temperature and flow cytometric measurements were performed immediately thereafter by FACSCanto flow cytometer (BD Biosciences, CA, USA). The mean value of red fluorescence intensity was divided by that of green fluorescence and expressed in fluorescence ratio (red/green) [29]. With respect to permeability, microbes were collected, washed and re-suspended in 10 mM sodium phosphate, pH 7.5, to an OD_600_ of 0.05. One μM SYTOX^®^ Green (Molecular Probes, OR, USA) was added into a 100 μL cell suspension in a dark 96-well plate for 30 min in the dark before addition of granulysin. The fluorescence intensity of SYTOX^®^ Green bound to cytosolic DNA was determined by a SpectraMax M2 microplate reader (Molecular Devices, CA, USA) with an excitation wavelength of 485 nm and an emission wavelength of 520 nm [30].

### Hydrophobicity Assay

A small volume of 8-Anilino-1-naphthalenesulfonic acid (ANS, Sigma Missouri, USA) solution at the concentrations as indicated (0 to 30 μM) was added to a 200 μL granulysin (6 μg/mL) in PBS in the dark at 20°C. The fluorescence of ANS was determined by a temperature-controlled FP-8500 fluorescence spectrophotometer (Jasco, Tokyo, Japan), which was excited at 370 nm and measured between 400 nm to 600 nm. The free form ANS exerts a maximum emission at 520 nm, however, it exhibits a blue shift of 470 nm once it is bound to hydrophobic protein [26].

### Biofilm formation assay and viability assay

One hundred μL of *P*. *aeruginosa* PAO1 (ca. 1×10^8^ cfu/mL) was distributed in flat-bottom 96-well microplate in the presence of granulysin in M63 minimal medium at 37°C for 24 hr. Subsequently, the planktonic cells were removed and the adherent biofilms were washed three times with water. One hundred and twenty five μL of 0.1% (w/v) crystal violet (Sigma, Missouri, USA) was added and incubated for 30 min. Excess crystal violet was removed and washed three times with water. The crystal violet remained in the biofilms were dissolved in 125 μL of 95% (v/v) ethanol and quantified by SpectraMax M2 microplate reader (Molecular Devices, CA, USA) [31]. Student’s *t*-test was used to evaluate the significant difference between biofilms with and without AMP treatment and a value of p<0.001 was considered significant. The viabilities of bacteria in the biofilms were assessed using LIVE/DEAD^®^ *Bac*Light^™^ Bacterial Viability Kit (Molecular Probes, OR, USA). Two hundred μL of *P*. *aeruginosa* PAO1 cell suspension (ca. 1×10^8^ cfu/mL) was distributed on chamber slides in the presence of granulysin at the concentration as indicated in M63 minimal medium at 37°C for 24 hr. The planktonic cells were removed and the adherent bacteria in the biofilm were washed three times with PBS and stained with two component dyes (SYTO 9 and propidium iodide in a 1:1 mixture) in PBS according to manufacturer’s instructions. The excitation/emission maxima for these two dyes were 480/500 nm for SYTO 9 live cell staining (green) and 490/635 nm for propidium iodide dead cell staining (red), respectively [31]. Fluorescence images were taken by LSM 700confocal laser-scanning microscope (Carl Zeiss, Jena, Germany).

## Results

### Preparation of soluble granulysin from *E*. *coli* having antimicrobial activity against *Pseudomonas aeruginosa*

The recombinant human 15 kDa granulysin fused with maltose-binding protein (MBP) was expressed in *E*. *coli* and purified to homogeneity by several column chromatographies (S1A–S1D Fig). The method was completely different from previously published reports which exploited the denaturation and renaturation of the protein from inclusion bodies [12, 19–21]. The actual molecular weights of 9 and 15 kDa granulysin were 4 and 6 Daltons less than those of predicted values according to their amino acid compositions, respectively (S1E Fig). These results indicate that the recombinant 9 kDa and 15 kDa granulysin may possess 2 and 3 pairs of disulfide bonds, respectively.

### Bactericidal activities of granulysin against *P*. *aeruginosa*

**Fig 1:**
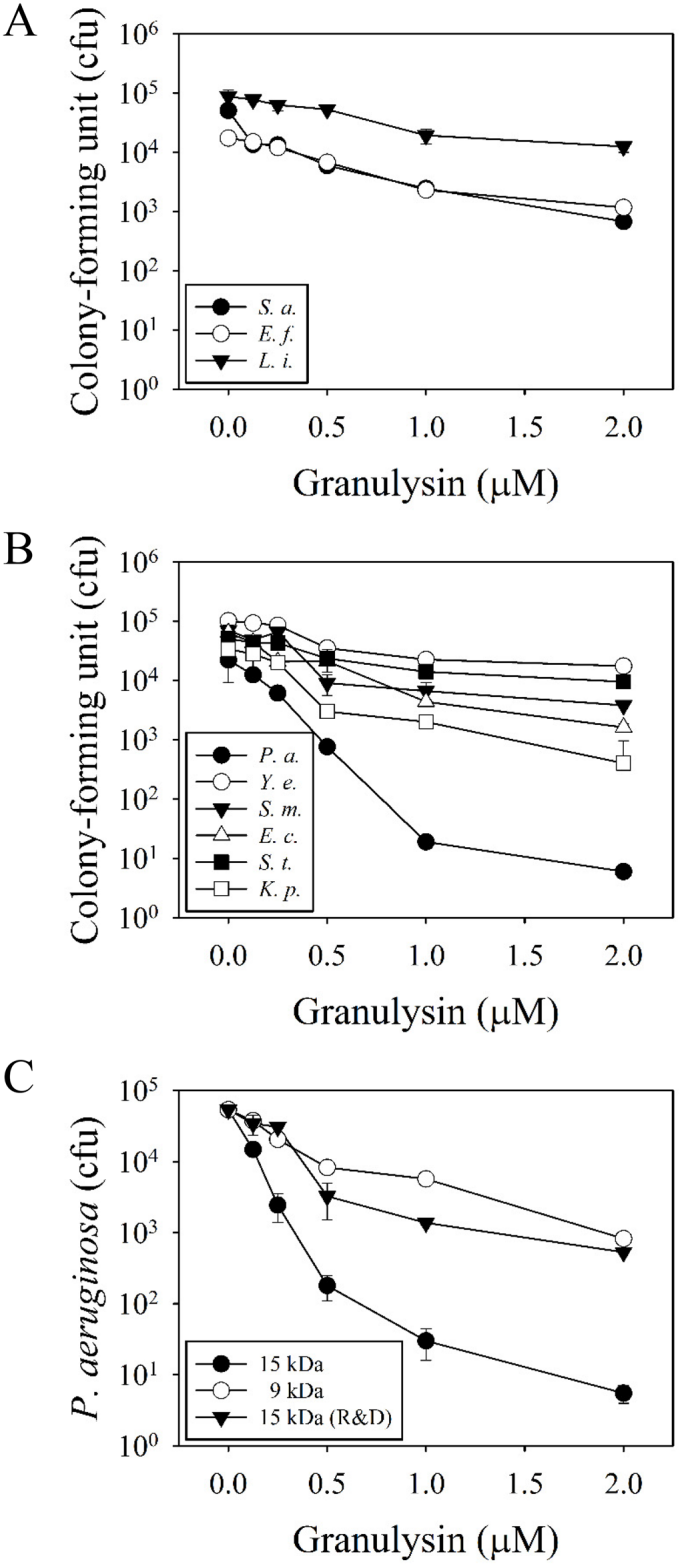
Bactericidal activities of granulysin. Antimicrobial activities of 15 kDa granulysin on Gram-positive (A) and Gram-negative (B) bacteria. Microbes (5–10×10^4^ cfu) were incubated with 15 kDa granulysin at 37°C in 10 mM sodium phosphate, pH 7.5, for 3 hr and plated on agar plates for the determination of remaining cfu. Comparison on the antimicrobial activity against *P*. *aeruginosa* among 15 kDa, 9 kDa granulysin and a commercial 15 kDa granulysin (R&D) were also employed (C). *S*. *a*., *Staphylococcus aureus*; *E*. *f*., *Enterococcus faecalis*; *L*. *i*., *Listeria innocua*; *P*. *a*., *Pseudomonas aeruginosa*; *Y*. *e*., *Yersinia enterocolitica*; *S*. *m*., *Serratia marcescens*; *E*. *c*., *Escherichia coli*; *S*. *t*., *Salmonella typhimurium*; *K*. *p*., *Klebsiella pneumonia*.

**Fig 2:**
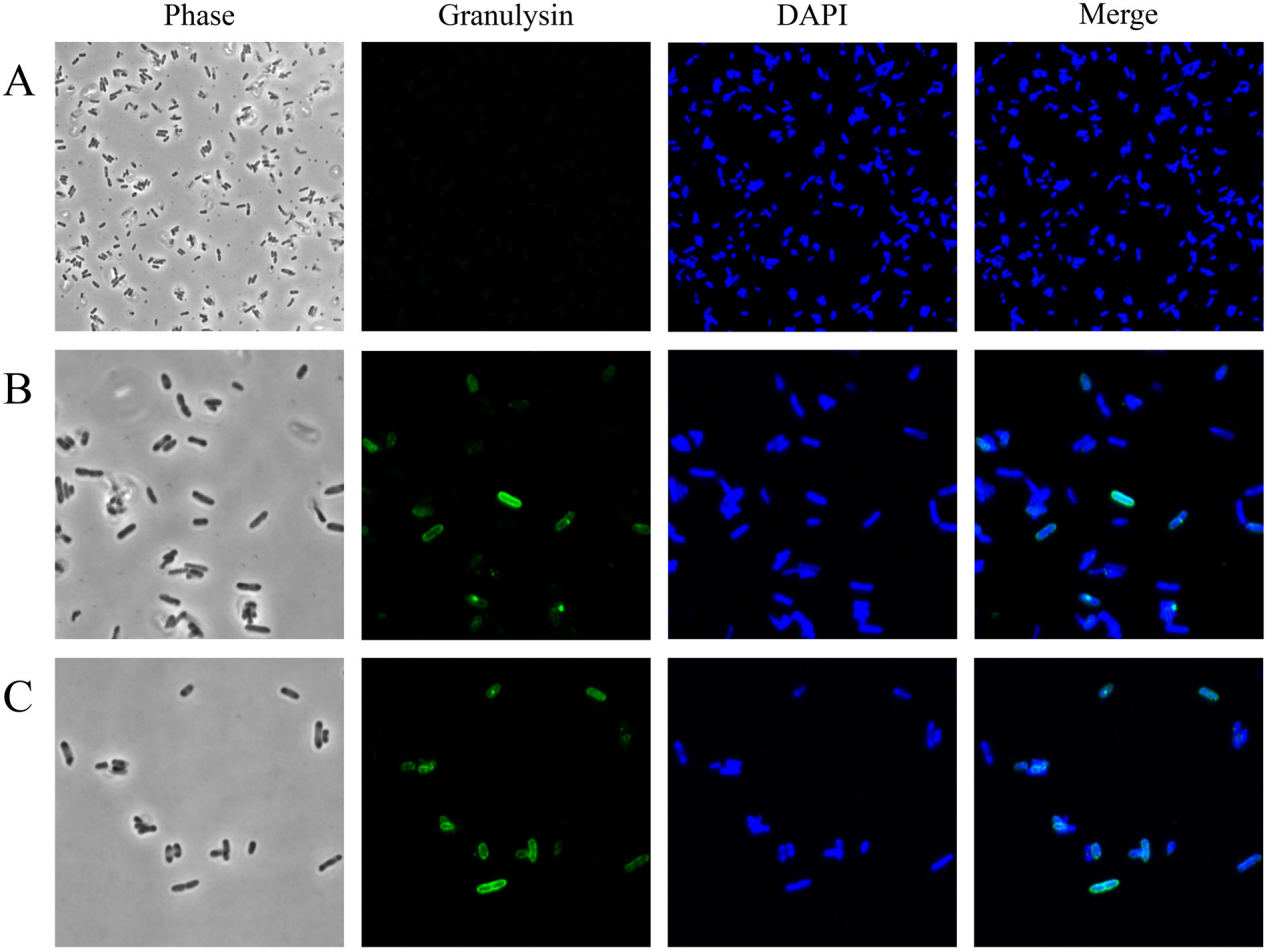
Localization of 15 kDa granulysin on *Pseudomonas aeruginosa* PAO1. Approximately 1×10^7^ microbes were incubated with 2 μM 15 kDa granulysin for 0 min (A), 60 min (B) and 180 min (C), respectively. Immunocytochemistry was performed using RF10 mAb for granulysin staining and DAPI for DNA staining. Images were taken using confocal laser-scanning microscopy (CLSM).

**Fig 3:**
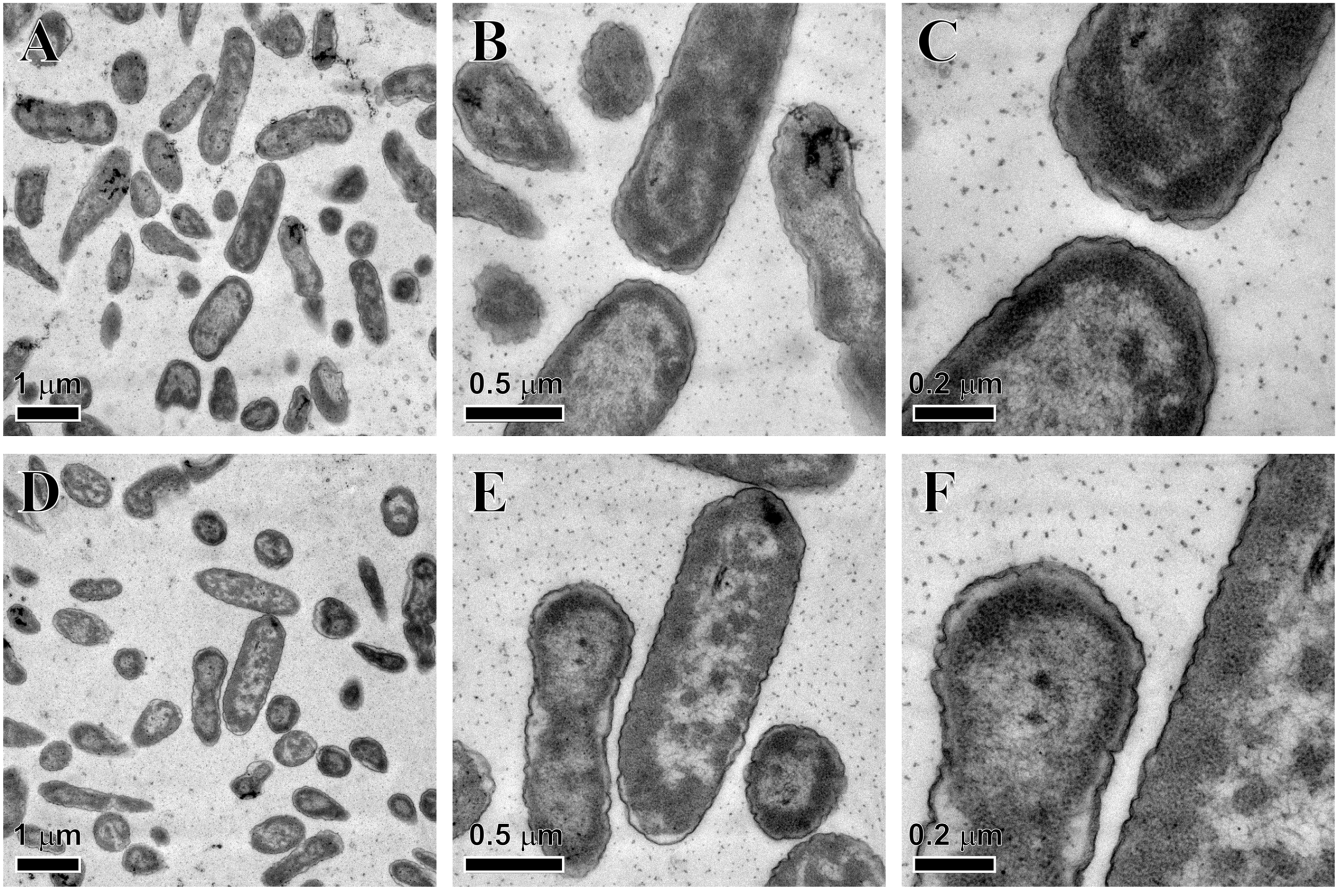
Morphology of *Pseudomonas aeruginosa* PAO1 after 15 kDa granulysin treatment. Approximately 1×10^7^ microbes were treated with 2 μM 15 kDa granulysin for 0 min (A-C) and 180 min (D-F) and observed by transmission electron microscopy (TEM).

Among the antimicrobial spectrum of 15 kDa granulysin, *P*. *aeruginosa* PAO1 was the most susceptible bacteria (10^4^-folds reduction in cfu at 2 μM), while the other bacteria either Gram-positive (*S*. *aureus*, *E*. *faecalis* and *L*. *innocua*) or Gram-negative (*Y*. *enterocolitica*, *S*. *marcescens*, *E*. *coli*, *S*. *typhimurium* and *K*. *pneumoniae*) were much less susceptible (Fig 1A and 1B). The 15 kDa granulysin was also much more effective than 9 kDa granulysin and the commercially-available 15 kDa granulysin (R&D) in the reduction of cfu (Fig 1C). The *P*. *aeruginosa* PAO1 showed resistance to the actions of ampicillin, kanamycin, and vancomycin (unpublished data). We found that the 15 kDa granulysin was only detected at the surface of bacteria, but not in the cytosol because a clearly green-fluorescence zone surrounding the DAPI-stained cytosol in blue was found even after 3 hr treatment (Fig 2). In contrast to most AMPs, the granulysin (2 μM) did not dramatically change the morphology of *P*. *aeruginosa* PAO1, by membrane blebbing/damage, cytoplasm condensation and component leakage as demonstrated by transmission electron microscopy (TEM) (Fig 3). These results suggest that granulysin binds to the bacterial surface and triggers a bactericidal pathway rather than entering the cytosol.

**Fig 4:**
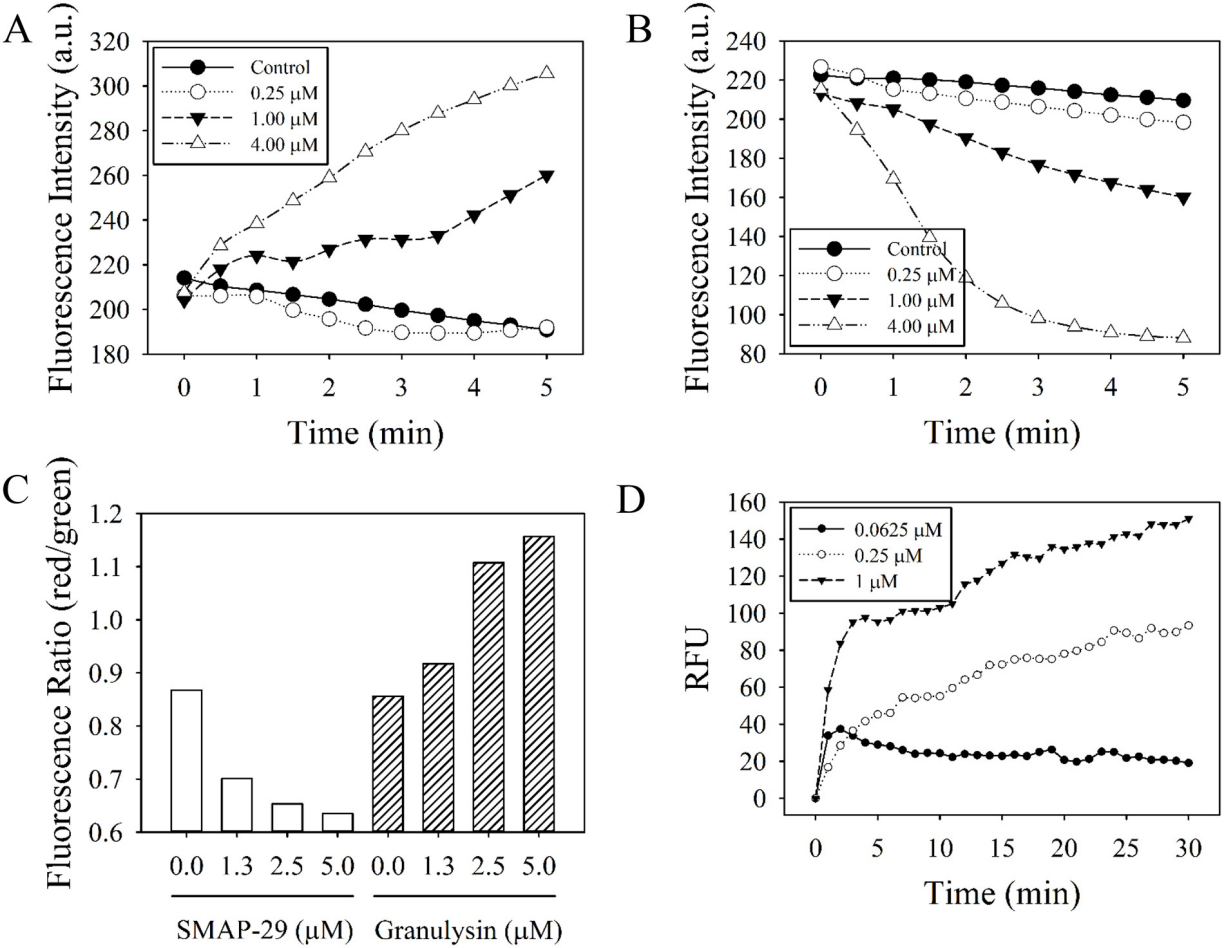
Effect of 15 kDa granulysin on the membrane potential and permeability of *Pseudomonas aeruginosa* PAO1. Membrane potential of SMAP-29- (A) and 15 kDa granulysin- (B) treated microbes were determined in the presence of DiSC_3_(5). Fluorescence intensity was monitored at an excitation wavelength of 622 nm and an emission wavelength of 670 nm. Data plotted are representative average values of three independent trials. a.u., absorbance unit. Membrane potential of treated microbes were also measured in the presence of DiOC_2_(3) by flow cytometry (C). Red/green fluorescence ratio was calculated using population mean fluorescence intensities for bacteria in the presence of SMAP-29 or 15kDa granulysin. Membrane permeability of microbes was monitored in the presence of SYTOX^™^ Green at 485 nm and 520 nm for excitation and emission wavelength, respectively (D). Data plotted are normalized with values of untreated sample and representative average values of three independent trials. RFU, relative fluorescence unit.

Similar to most AMPs, the sheep myeloid antimicrobial peptide, SMAP-29, induced depolarization of bacterial membrane potential because the DiSC_3_(5) dye was released into the surrounding medium that caused the increase of fluorescence intensity in a dose-dependent manner (Fig 4A). However, the 15 kDa granulysin hyperpolarized the membrane potential due to fluorescent intensity decrease after granulysin treatment (Fig 4B). The depolarization of membrane potential by SMAP-29 and hyperpolarization by 15 kDa granulysin were further confirmed by another membrane potential indicator DiOC_2_(3) using flow cytometry. The emission of fluorescence shifted to red as the dye molecules aggregate at higher cytosolic concentrations under higher membrane potentials caused by 15 kDa granulysin, while it shifted to green by SMAP-29 (Fig 4C). The membrane permeability of the bacteria markedly increased a few minutes after granulysin treatment (1 μM) as a result of the green fluorescent SYTOX^®^ Green being internalized into the cytosol and complexed with bacterial DNA (Fig 4D). Taken together, these results demonstrate that granulysin effectively hyperpolarizes membrane potential, increases membrane permeability, and activates the bactericidal pathway.

### Components of sera affecting bactericidal activity of granulysin

**Fig 5:**
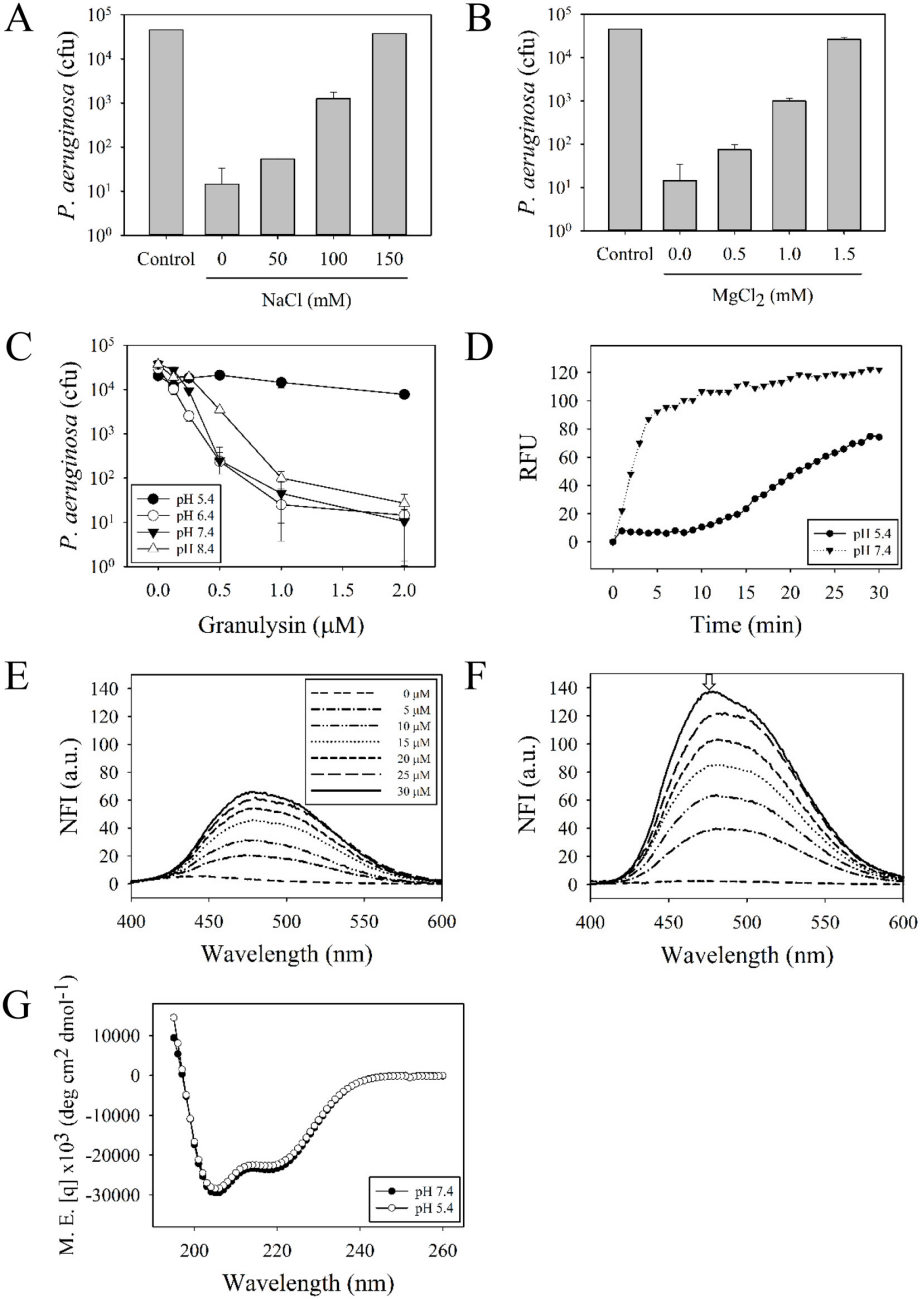
Effect of cations and pH on the antimicrobial activity, membrane permeability against *Pseudomonas aeruginosa* PAO1 and secondary structure of 15 kDa granulysin. The antimicrobial activities of 2 μM 15 kDa granulysin were determined in the presence of NaCl (A) and MgCl_2_ (B) at the concentrations indicated. Microbes were incubated with granulysin at different pH values and the remaining colony-forming units were determined (C). SYTOX^™^ Green was added to monitor the change in bacterial membrane permeability in the presence of 1 μM granulysin at pH 5.4 and pH 7.4, respectively (D). Fluorescence emission spectra of 8-Anilino-1-naphthalenesulfonic acid (ANS) was excited at 370 nm and measured between 400 and 600 nm in the presence of 1 μM granulysin at pH 7.4 (E) and pH 5.4 (F). Arrow indicates the blue shift (470 nm) of emission spectra of ANS-granulysin hydrophobic complex. Data plotted are normalized by the fluorescence intensity of ANS alone at pH 7.4 or pH 5.4, respectively. NFI, normalized fluorescence intensity. Circular dichroism spectrum of 20 μM 15 kDa granulysin in 20 mM HEPES, 50 mM NaCl, pH 7.4 or pH 5.4 (G). M.E., molar ellipticity.

To determine whether the antimicrobial activity of granulysin is still effective in body fluids, other tissues, or affected by components in sera, we examined the factors such as salts, divalent cations and pH values. The results showed that the antimicrobial activity was repressed by 150 mM NaCl or 1.5 mM MgCl_2_, which is similar to the concentrations in sera (125 mM for NaCl and 1.25 mM for MgCl_2_) (Fig 5A and 5B). In addition, the granulysin retained its antimicrobial activity close to neutral pH values (6.4 to 8.4), but was inactive at pH 5.4 (Fig 5C). The increase in membrane permeability was also repressed in the acidic environment (pH 5.4) (Fig 5D). These results suggest that granulysin may exert its function in a neutral environment that lacks body fluids. To further investigate the possible effects of an acidic environment on the structure of granulysin, the changes of hydrophobicity were measured by the emission spectra of a hydrophobic fluorescent dye 8-anilino-1-naphthalenesulfonate (ANS). The ANS exhibited an emission maximum at 520 nm in free form at pH 7.4, and a blue shift to 470 nm once it binds to the hydrophobic granulysin at pH 5.4 (Fig 5E and 5F). However, the CD spectra of 15 kDa granulysin in pH 7.4 and 5.4 showed that they possess almost identical secondary structures (Fig 5G). These results suggest that an acidic environment renders granulysin hydrophobic and reduces the bactericidal activity without changing the secondary structure.

### Inhibition of biofilm formation by granulysin

**Fig 6:**
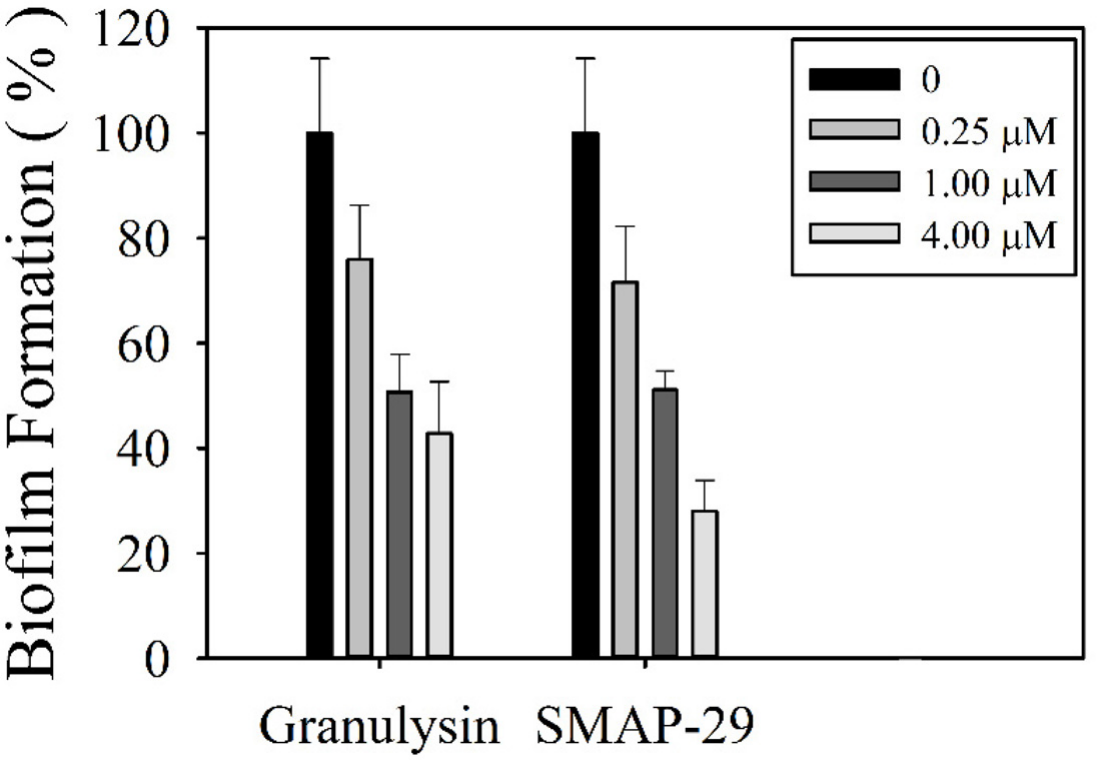
Effect of 15 kDa granulysin on the biofilm formation of *Pseudomonas aeruginosa* PAO1. Microbes were grown in M63 minimal media in the presence of SMAP-29 and 15 kDa granulysin at 37°C for 24 hr and quantified by crystal violet staining and measured at the absorbance of 600 nm. Error bars represent the standard errors of the means. The asterisks indicate samples that are significantly different from control. (Student’s t-tests; p≤0.01).

**Fig 7:**
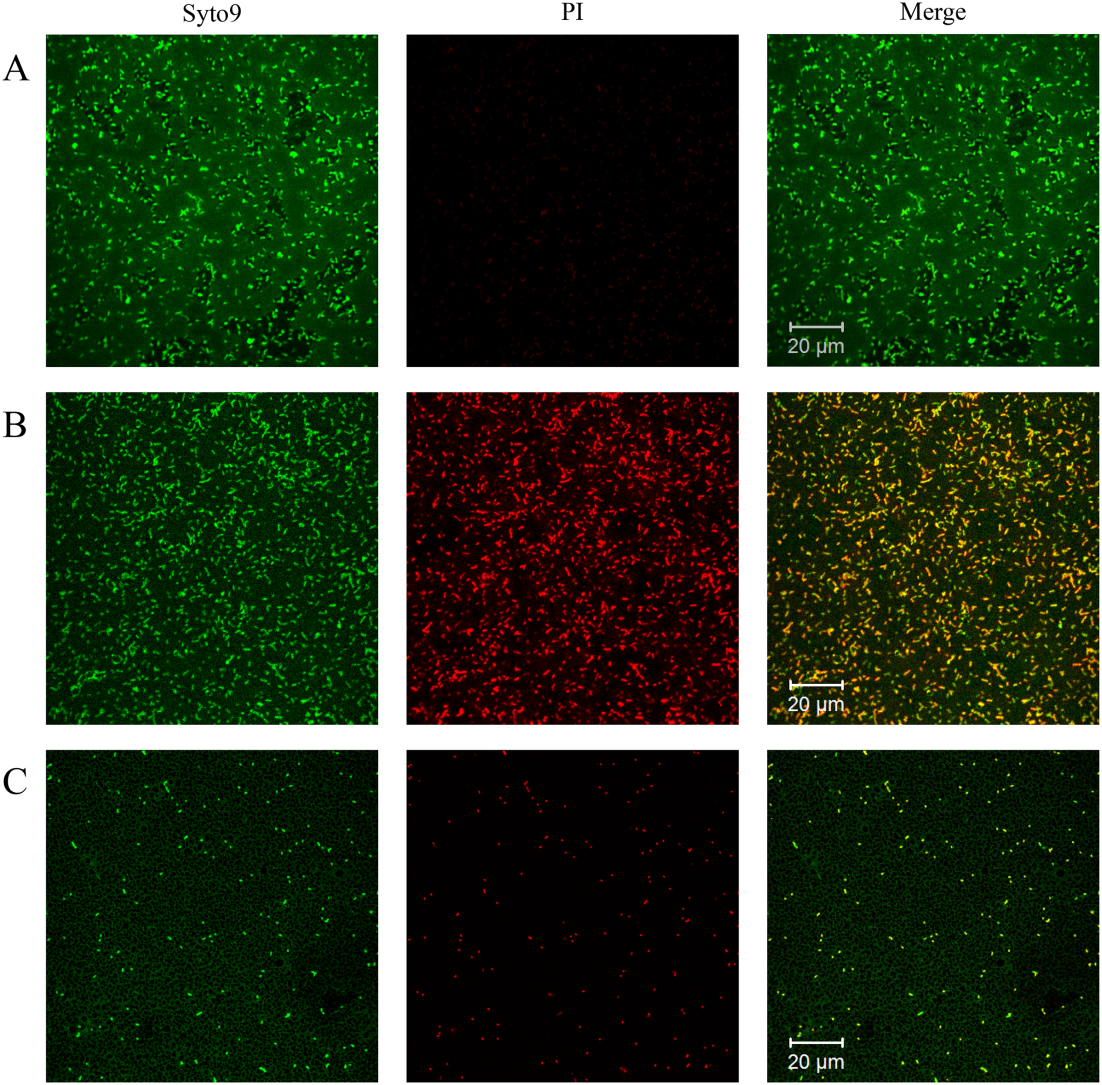
Viability of bacteria in the biofilm of *Pseudomonas aeruginosa* PAO1 after 15 kDa granulysin treatment. Microbes were visualized by staining with SYTO-9 (green fluorescence for living bacteria) and propidium iodide (red fluorescence for dead bacteria) in the presence of 0 (A), 0.25 μM (B), and 4 μM granulysin (C) at 37°C for 24 hr. Images were obtained by confocal laser scanning microscope (CLSM).

To further evaluate the efficacy of granulysin in the treatment of bacterial infection, the effect of 15 kDa granulysin on the formation of biofilm was examined where the bacteria can be protected from actions of antibiotics and AMPs. Both 15 kDa granulysin and SMAP-29 markedly reduced biofilm formation by *P*. *aeruginosa* in M63 minimal medium in a dose-dependent manner (Fig 6). The *P*. *aeruginosa* PAO1 was able to grow in a static state and form a homogenous biofilm on chamber slides for 24 hr with robust viability. However, there remained a homogenous biofilm but with less viability in the presence of 0.25 μM granulysin as visualized by CLSM using SYTO-9 and propidium iodide to stain live and dead cells, respectively (Fig 7A and 7B). The remaining bacteria, however, were unable to establish a homogenous biofilm and lost the ability to colonize surfaces in the presence of 4 μM granulysin (Fig 7C). By contrast, 15 kDa granulysin had no effect on the viability of one day-old preformed biofilms (data not shown). These data demonstrate that granulysin is not only able to kill planktonic bacteria but also inhibits biofilm formation under the doses employed as demonstrated by crystal violet staining (Fig 6A and 6B).

## Discussion

In addition to antimicrobial activity, granulysin plays important roles in tissue transplantation and immune-mediated skin diseases [11, 32]. The 15 kDa granulysin has been shown to be responsible for disseminated keratinocyte death in SJS/TEN [16]. It is also significantly elevated and correlate with the severity of GVHD [17].

Progress in research on the cytotoxic mechanisms of granulysin has been hindered by the lack of protein abundance. Currently, the 15 kDa granulysin products are available for purchase from two companies, R&D Systems and Novus Biologicals (Abnova). The protein from R&D Systems contains a 10-histidine tag at the C-terminus and the other from Novus Biologicals contains a glutathione S-transferase (GST) tag at the N-terminus. These added tags may affect their function [25]. Insect cell systems and cell-free *E*. *coli* expression systems have also been employed to express the 15 kDa granulysin, but with high cost and poor yield [16, 25]. Alternatively, both 9 and 15 kDa granulysins can be collected from the culture media of *Pichia pastoris* after secretion, and followed by purification using affinity and cation exchange chromatography. The downside is that the bactericidal activity of 15 kDa granulysin (50–100 μM) is much less effective than that of the 9 kDa granulysin (1–2 μM) [33]. Productions of recombinant cytotoxic protein are usually prepared by the denaturation/refolding of insoluble proteins in the inclusion bodies of *E*. *coli*. However, the yield can be limited, the procedure is time-consuming and the antimicrobial activity is low (10–50 μM) [13, 19, 22]. In this study, we successfully constructed an expression vector which contains His×6, maltose binding protein (MBP), PreScission^™^ protease recognition site, 9/15 kDa granulysin, and expressed the fusion protein in *E*. *coli*. The soluble granulysin was released from MBP-fused protein by the PreScission^™^ protease and purified by a few column chromatography steps. The merit of this study is that no protein denaturation/refolding, animal cell culture and *in vitro* cell-free synthesis were employed. The recombinant 15 kDa granulysin possesses strong antimicrobial activity against *P*. *aeruginosa* at 2 μM.

Both the 9 kDa granulysin refolded from *E*. *coli* inclusion bodies, and the 15 kDa granulysin collected from *P*. *pastoris* culture media exerted broad antimicrobial spectra, but showed low activity against Gram-positive, -negative bacteria and fungi, like *S*. *aureus*, *L*. *monocytogenes*, *E*. *coli*, *S*. *typhimurium* and *Candida albicans* at 10–50 μM [19–21, 33]. Interestingly, the soluble 15 kDa granulysin prepared from MBP-fused protein in this study exerted strong and specific antimicrobial activity against *P*. *aeruginosa*. This may suggest that some specific receptors on *P*. *aeruginosa* may be responsible for the recognition of granulysin. The outer membrane lipoprotein OprI of *P*. *aeruginosa* is responsible for the recognition of cationic α-helical antimicrobial peptide [26], but it was not recognized by the 15 kDa granulysin because the bactericidal activity of granulysin was not repressed by the presence of excess amounts of recombinant OprI or anti-OprI antibodies (data not shown). *O*-linked glycosylation was found in both 9 and 15 kDa granulysin obtained from the secreted culture media of *P*. *pastoris*, but it was not required for their antimicrobial activities [33]. The molecular weights of 9 and 15 kDa granulysin prepared from *E*. *coli* were 8800 and 14952 Daltons, respectively. They were 4 and 6 Daltons less than those of predicted values according to their amino acid sequences. No glycosylation occurred in both forms (S1E Fig). Our results indicate that the recombinant 9 kDa granulysin possesses 2 pairs of disulfide bonds, which is in good agreement with the crystal structure of 9 kDa granulysin, Cys54-Cys117 and Cys81-Cys92 [34]. The 15 kDa granulysin is shown to be tethered by 3 pairs of disulfide bonds, Cys28/Cys30-Cys123, Cys54-Cys117 and Cys81-Cys92.

Depolarization or disruption of cytoplasmic membrane renders the bound fluorescent dye DiSC_3_(5) to be released into medium leading to increased intensity of fluorescence [35]. Similar to most AMPs, the sheep myeloid antimicrobial peptide (SMAP-29) exhibited membrane potential depolarization (Fig 4A) once the bacterial membrane was permeabilized or disrupted [30]. On the contrary, the 15 kDa granulysin hyperpolarized membrane potential in a dose-dependent manner detected by fluorescence spectrometry and flow cytometry using different dye indicators, DiSC_3_(5) and DiOC_2_(3), as shown in Fig 4B and 4C. Although membrane potential depolarization is considered to be an initial event of membrane injury, the hyperpolarization has also been reported as an adaptation related to bacterial viability [24, 29, 36, 37]. Hyperpolarization has also been associated with the formation of superoxide radicals [37], which are implicated in membrane integrity and cell viability [24, 29]. The hyperpolarization of membrane potential induced by the 15 kDa granulysin indicates that it may have a novel bactericidal mechanism which is different from those of conventional AMPs.

The AMPs exert host defense activity against invading pathogens in various environments containing salts, divalent cations, serum proteins and changes in pH. Here, we found the antimicrobial activities of granulysin against *P*. *aeruginosa* to be greatly inhibited by the presence of divalent cations or by the acidic environment at pH 5.4 (Fig 5A–5C). The pH values in living organisms usually vary with tissues such as the neutrophil phagolysosomes or inflammatory condition-induced acidic pH values between 4.5 and 6.5 [38]. On the other hand, the bloodstream and mucosal surfaces maintain neutral pH values of 7.2 to 7.5 [39]. The antimicrobial activities of human β-defensin-3 and LL-37 against *P*. *aeruginosa* at acidic pH values are less effective than neutral environments [40]. Our results reveal that granulysin may exhibit its bactericidal activity only in neutral environments devoid of body fluids such as the skin.

Amphipathicity of an AMP including hydrophobicity and net charge is crucial for its antimicrobial activity [41, 42]. The hydrophobicity of an amphipathic peptide facilitates its interaction with bacterial hydrophobic membrane. However the positive charges of AMPs promote selectivity for negatively charged components on microbial surfaces. Both properties are essential for its function. The hydrophobicity of a peptide is increased *in vitro* if the environment becomes hydrophobic, as in the presence of 30~50% trifluoroethanol [43]. The antimicrobial activity of a peptide against *P*. *aeruginosa* varies with the extent of hydrophobicity in certain ranges [44, 45]. Our previous results demonstrated that α-helical cationic antimicrobial peptide, SMAP-29, was able to interact with the bacterial outer membrane receptor protein, OprI, and to render it hydrophobic to induced membrane fusion [26]. Interestingly, we found that both membrane permeability and antimicrobial activity of 15 kDa granulysin were markedly inhibited in an acidic environment (pH5.4), which increased the hydrophobicity of granulysin as shown in Fig 5C–5F. These results suggest that the electrostatic interactions between amphipathic AMPs and specific receptors are important prior to fusion with bacterial outer membrane through the hydrophobic sides of AMP.

The 9 kDa granulysin is cytotoxic to tumor cells through the apoptotic pathway by the release of cytochrome *c* and apoptosis-inducing factor (AIF), and also by activation of caspases and endonucleases [46–50]. However, the cytotoxicity of the 15 kDa granulysin remains controversial. Chung *et al*. showed that the 15 kDa granulysin, but not 9 kDa granulysin, in blister fluids of patients with SJS/TEN can exert cytotoxic activity against keratinocyte that leads to the severity of the skin-associated disease [16]. However, Clayberger *et al*. demonstrated that the 15 kDa granulysin from insect cells can induce the differentiation from monocytes to immature dendritic cells (iDC) but without cytotoxicity in U937 tumor cells [23]. The discrepancy in cytotoxicity may be due to the following reasons. First, granulysin may possess selective cytotoxicity toward different cell types. Second, granulysin needs the aid of other components such as perforin, sFasL, and granzyme B, to exert the cytotoxic events although it is the key mediator of cytotoxicity [16].

In this study, we provide a simple way to express and purify soluble 15 kDa granulysin which exhibited specific antimicrobial activity against *P*. *aeruginosa*. The 15 kDa granulysin hyperpolarized membrane and permeated the membrane through a novel mechanism to kill bacteria which is different from most AMPs. Our results render the study possible for the action mechanisms of antimicrobial activity and cytotoxicity to different human cell types in transplantation and skin-associated diseases.

## Supporting Information

S1 Fig Expression and purification of granulysin. FPLC chromatographies of 15 kDa granulysin by HisTrap^™^ HP (A), Superose^™^ 12 (B), and HiTrap^™^ SP FF (C) column and analysis of proteins from column eluates by 15% SDS-PAGE and Coomassie Blue staining (D). *Lane 1*, crude lysate; *Lane 2*, eluates of phosphate cellulose (P11) chromatography; *Lane 3*, eluates of HisTrap^™^ HP column chromatography; *Lane 4*, digestion product of PreScission^™^ Protease; *Lane 5*, eluates of Superose^™^ 12 gel filtration chromatography; *Lane 6*, eluates of HiTrap^™^ SP FF column chromatography. Arrow indicates the purified 15 kDa granulysin. The molecular masses of recombinant 9 and 15 kDa granulysin were determined by ESI-MS spectra (E).
(PDF)
