# Supplementary material for: Antimicrobial Properties of an Immunomodulator - 15 kDa Human Granulysin
Source: PLoS One. 2016 Jun 8;11(6):e0156321. doi: 10.1371/journal.pone.0156321 (PMC4898823; doi:10.1371/journal.pone.0156321)
Supplement: S1 Fig — FPLC chromatographies of 15 kDa granulysin by HisTrap™ HP (A), Superose™ 12 (B), and HiTrap™ SP FF (C) column and analysis of proteins from column eluates by 15% SDS-PAGE and Coomassie Blue staining (D). Lane 1, crude lysate; Lane 2, eluates of phosphate cellulose (P11) chromatography; Lane 3, eluates of HisTrap™ HP column chromatography; Lane 4, digestion product of PreScission™ Protease; Lane 5, eluates of Superose™ 12 gel filtration chromatography; Lane 6, eluates of HiTrap™ SP FF column chromatography. Arrow indicates the purified 15 kDa granulysin. The molecular masses of recombinant 9 and 15 kDa granulysin were determined by ESI-MS spectra (E). (PDF) [file pone.0156321.s001.pdf]

S1 Fig.

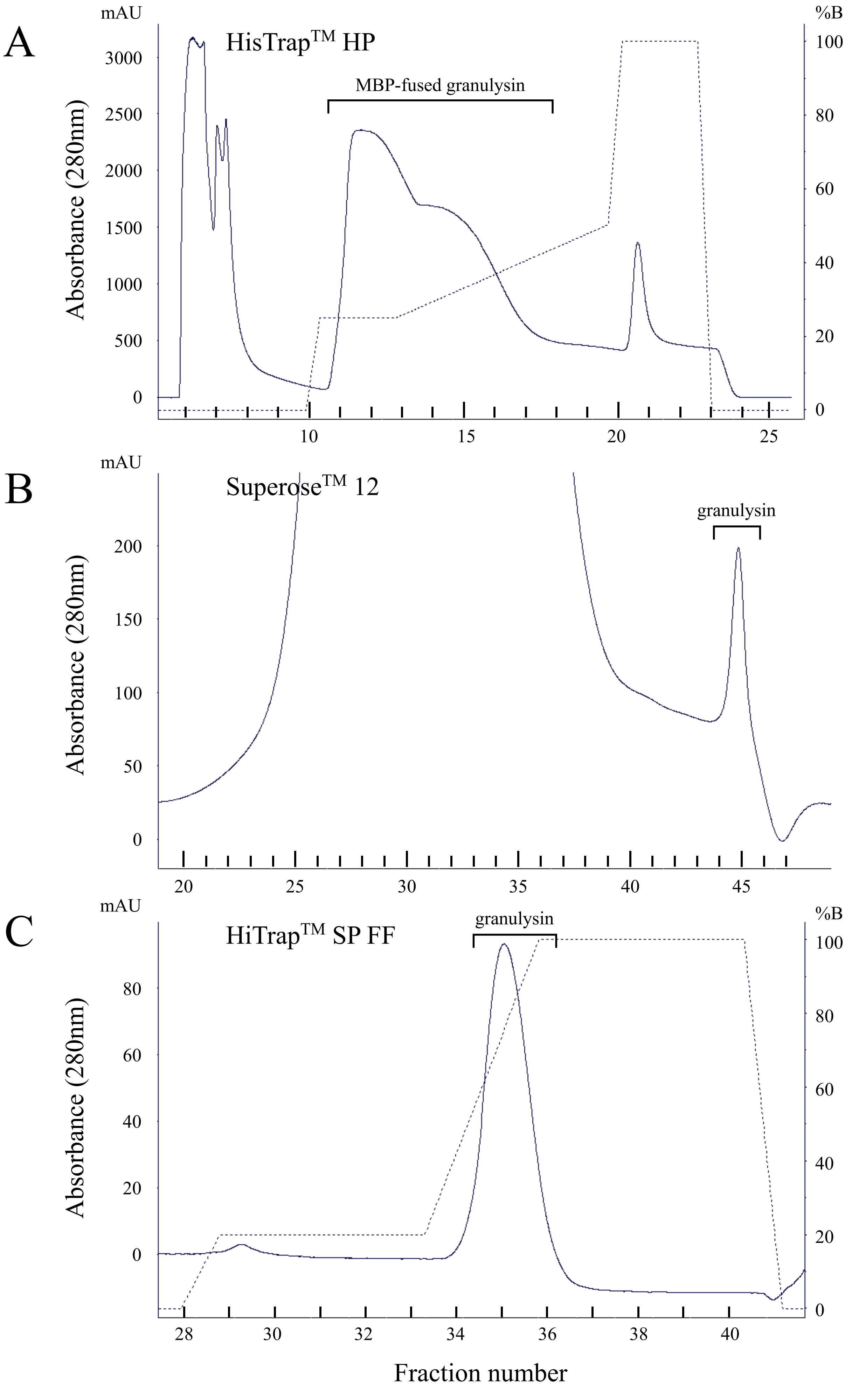
A
HisTrap™ HP
MBP-fused granulysin
mAU
%B
Absorbance (280nm)
3000
2500
2000
1500
1000
500
0
100
80
60
40
20
0
10
15
20
25
B
Superose™ 12
granulysin
mAU
Absorbance (280nm)
200
150
100
50
0
20
25
30
35
40
45
C
HiTrap™ SP FF
granulysin
mAU
%B
Absorbance (280nm)
80
60
40
20
0
100
80
60
40
20
0
28
30
32
34
36
38
40
Fraction number

D

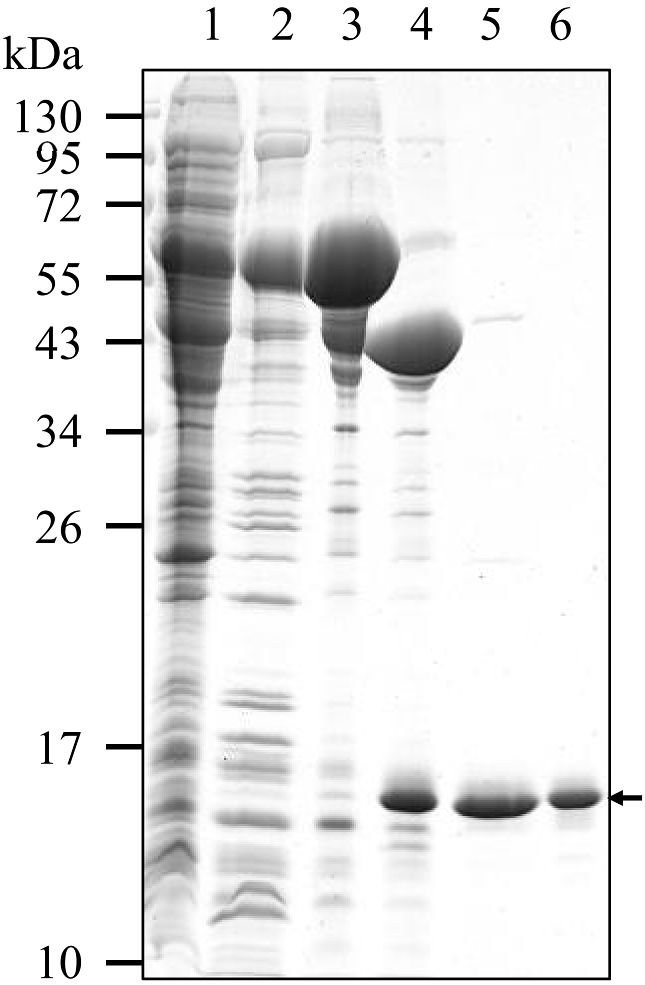

E

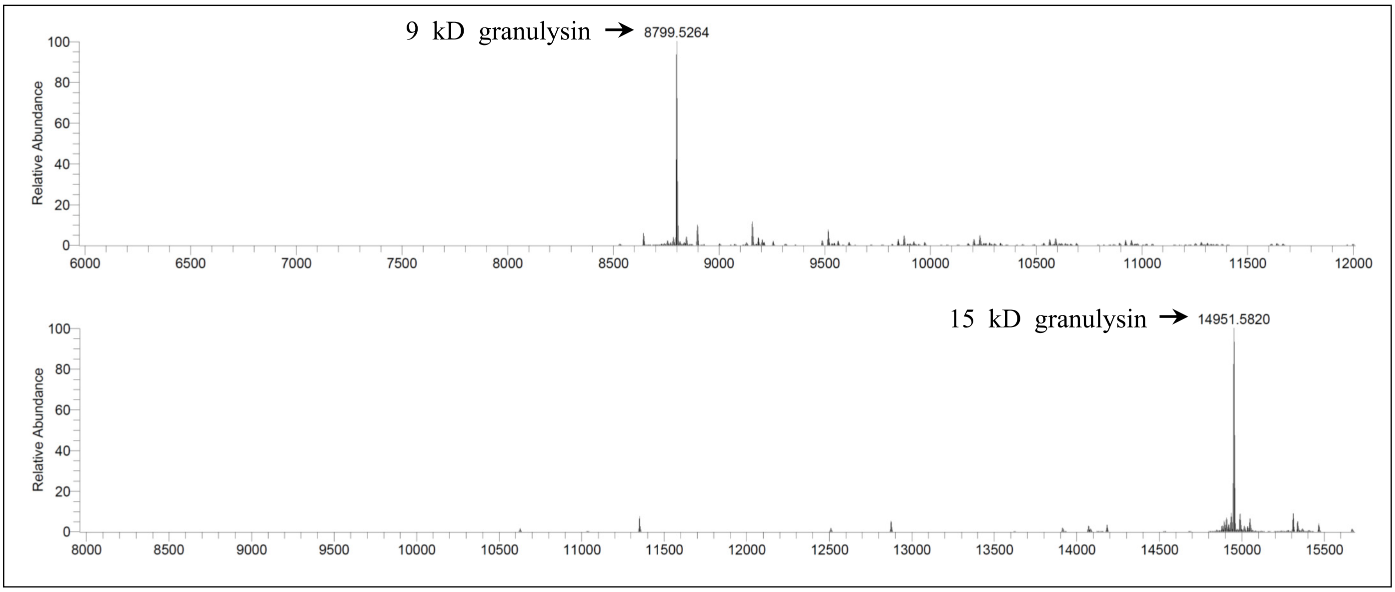

Molecular weight

S1 Fig. Expression and purification of granulysin. FPLC chromatographies of 15 kDa granulysin by HisTrap^{TM} HP (A), Superose^{TM} 12 (B), and HiTrap^{TM} SP FF (C) column and analysis of proteins from column eluates by 15% SDS-PAGE and Coomassie Blue staining (D). *Lane 1*, crude lysate; *Lane 2*, eluates of phosphate cellulose (P11) chromatography; *Lane 3*, eluates of HisTrap^{TM} HP column chromatography; *Lane 4*, digestion product of PreScission^{TM} Protease; *Lane 5*, eluates of Superose^{TM} 12 gel filtration chromatography; *Lane 6*, eluates of HiTrap^{TM} SP FF column chromatography. Arrow indicates the purified 15 kDa granulysin. The molecular masses of recombinant 9 and 15 kDa granulysin were determined by ESI-MS spectra (E).
